# Neuropsychology of posteromedial parietal cortex and conversion factors from Mild Cognitive Impairment to Alzheimer’s disease: systematic search and state-of-the-art review

**DOI:** 10.1007/s40520-021-01930-y

**Published:** 2021-07-07

**Authors:** Ciro Rosario Ilardi, Sergio Chieffi, Tina Iachini, Alessandro Iavarone

**Affiliations:** 1 Department of Psychology, University of Campania “Luigi Vanvitelli”, Caserta, Italy; 2 Department of Experimental Medicine, University of Campania “Luigi Vanvitelli”, Naples, Italy; 3 Laboratory of Cognitive Science and Immersive Virtual Reality, Department of Psychology, University of Campania “Luigi Vanvitelli”, Caserta, Italy; 4 Neurological Unit, CTO Hospital, AORN “Ospedali Dei Colli”, Naples, Italy

**Keywords:** Posteromedial parietal cortex, Mild cognitive impairment, Conversion, Visuospatial working memory, Anosognosia for memory deficits, Visuomotor control

## Abstract

In the present review, we discuss the rationale and the clinical implications of assessing visuospatial working memory (VSWM), awareness of memory deficits, and visuomotor control in patients with mild cognitive impairment (MCI). These three domains are related to neural activity in the posteromedial parietal cortex (PMC) whose hypoactivation seems to be a significant predictor of conversion from MCI to Alzheimer’s disease (AD) as indicated by recent neuroimaging evidence. A systematic literature search was performed up to May 2021. Forty-eight studies were included: 42 studies provided analytical cross-sectional data and 6 studies longitudinal data on conversion rates. Overall, these studies showed that patients with MCI performed worse than healthy controls in tasks assessing VSWM, awareness of memory deficits, and visuomotor control; in some cases, MCI patients’ performance was comparable to that of patients with overt dementia. Deficits in VSWM and metamemory appear to be significant predictors of conversion. No study explored the relationship between visuomotor control and conversion. Nevertheless, it has been speculated that the assessment of visuomotor abilities in subjects at high AD risk might be useful to discriminate patients who are likely to convert from those who are not. Being able to indirectly estimate PMC functioning through quick and easy neuropsychological tasks in outpatient settings may improve diagnostic and prognostic accuracy, and therefore, the quality of the MCI patient’s management.

## Introduction

Mild cognitive impairment (MCI) is a clinical syndrome characterized by a moderate cognitive decline in the absence of a significant impact on functional autonomies. In many cases, MCI represents the prodromal stage of a major neurocognitive disorder. According to the diagnostic algorithm proposed by Petersen et al. in their first report [1] and further revisions [2, 3], there are four MCI subtypes: amnestic MCI–single domain (aMCI), amnestic MCI–multiple domain (aMCI-md), non-amnestic MCI–single domain (naMCI), and non-amnestic MCI–multiple domain (naMCI-md). The diagnosis of aMCI is postulated in the presence of a selective memory disorder, referred by the patient and confirmed by an informant (a relative or the General Practitioner), and attested by a score below the normal range for age and education at memory testing. The aMCI patient does not exhibit impairment in activities of daily living nor signs of overt dementia. When the memory deficit is accompanied by impairment in at least one other cognitive domain (e.g., language, executive functions, visuospatial skills), the diagnosis of aMCI-md is justified. Diagnosis of naMCI is advanced when a single non-memory domain is impaired, whereas naMCI-md refers to impairment in multiple non-memory domains [3].

The annual rate of conversion from MCI to dementia varies between 8 and 15% [4]. The aMCI and aMCI-md subtypes appear to be the neuropsychological profiles most frequently associated with the onset of Alzheimer’s disease (AD) and vascular dementia (VaD) [5, 6], while patients with non-amnestic MCI are more likely to develop dementia with Lewy body (LBD) [7] or frontotemporal dementia (FTD) [6]. However, data about rates of conversion from each clinical MCI phenotype to different forms of dementia are highly variable.

The diagnosis of MCI acquires prognostic significance when a pathognomonic symptom, e.g., a subclinical memory deficit, is accompanied by neurobiological evidence such as cortical thinning, imbalances in the concentration of Aβ-42 and tau protein in the cerebrospinal fluid, or detection of hypometabolic clusters [8]. In this respect, a recent meta-analysis [9] including nine ^18^Fuorodeoxyglucose positron emission tomography (^18^F-FDG-PET) studies compared 93 aMCI patients converted with 129 aMCI patients non-converted to AD. Results showed that hypometabolism in the posteromedial parietal cortex (PMC) at baseline, with particular reference to the posterior cingulate cortex and precuneus, represented a robust biomarker of progression to AD [9]. Other studies generalized this finding to different MCI subtypes [10, 11].

Some studies have suggested that a diagnostic approach combining both neuroimaging techniques and neuropsychological assessment may improve the accuracy in discriminating MCI patients who will later convert from those who will not [12–14]. However, it is both logistically and financially challenging to subject all eligible patients to imaging examination. If an abnormal activity in some PMC regions is predictive of progression to AD, the administration of neuropsychological tests assessing the integrity of the PMC neurocognitive correlates may be useful for “sifting through” the patients and improving diagnostic and prognostic markers.

**Fig. 1:**
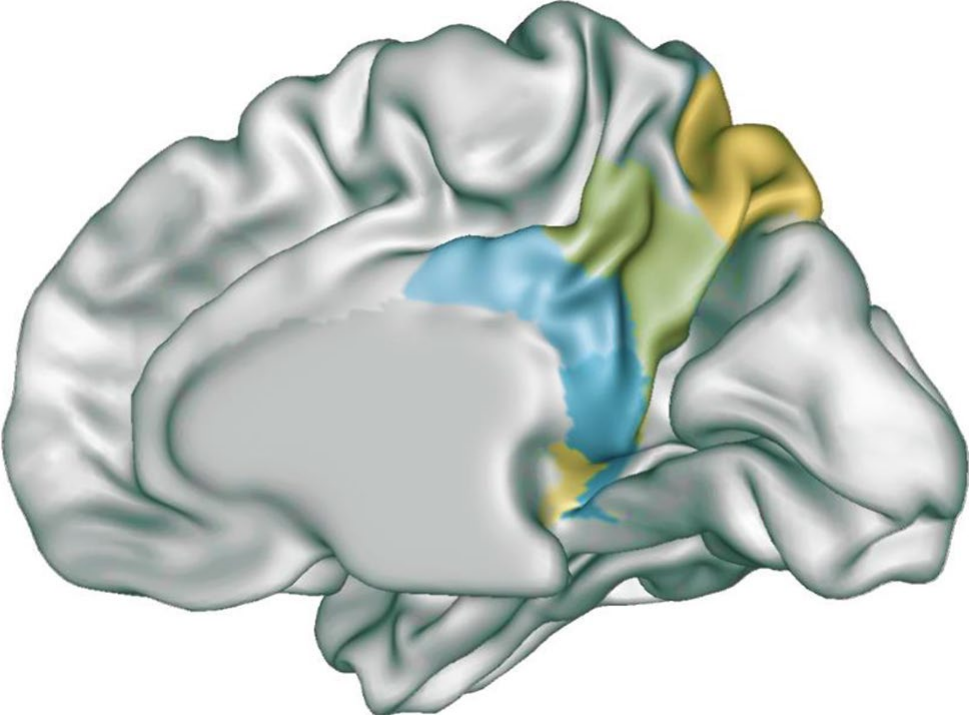
Architecture of PMC. From the top-down, the figure shows the precuneus (BA7 and BA31), the posterior cingulate cortex (BA23), and the retrosplenial cortex (BA30 and BA29). Starting from individual standard three-dimensional brain models provided by Brain Tutor 3D software, the figure was constructed by overlaying multiple models

The PMC is an architectonically discrete region comprising the precuneus (BA7 and BA31), the posterior cingulate (BA23) and the retrosplenial cortex (BA30 and BA29). The cytoarchitectonic areas of PMC are strongly interconnected (Fig. 1). The precuneus extends medially in correspondence of BA7 and 31, also occupying most of the lateral parietal cortex above the intraparietal sulcus. It shows intimate interconnections with the adjacent posterior cingulate and retrosplenial cortices and has anatomofunctional relationships with other parietal areas, namely, the caudal parietal operculum, the inferior and superior parietal lobules, and the inferior parietal sulcus. Additionally, its main extra-parietal projections lead into some frontal areas including the anterior cingulate cortex, premotor cortex, and dorsolateral prefrontal cortex [15].

The precuneus and other areas of PMC, in synergy with the frontal cortex, are mainly recruited during tasks involving episodic memory [15, 16], visuospatial working memory (VSWM) [17, 18], awareness of memory deficits [16, 19–21], and visuomotor abilities [15, 22–24]. Early deficits embracing episodic memory are widely acknowledged in MCI syndrome, and in particular for the amnestic variant. Therefore, common screening protocols already include valid and reliable psychometric measures of episodic memory. For this reason, discussing the implications for the assessment of episodic memory would be pleonastic. Conversely, other domains depending on PMC, i.e., VSWM, metamemory, and visuomotor control, are mostly neglected in clinical practice. Their role in improving the diagnosis of MCI and in predicting disease progression deserves more extensive investigation.

This review aims at (a) merging the available data linking the PMC neurocognitive correlates to the conversion from MCI towards AD, and (b) suggesting a number of tools easily usable in the outpatient clinical practice for monitoring the integrity of the above-mentioned domains. To date, neuropsychological “gold standards” ubiquitously accepted by the scientific community are missing.

## Materials and methods

### Search strategy and eligibility criteria

We performed a systematic search in PubMed with no years restriction. The string (“mild cognitive impairment” OR “mci” OR “mild dementia” OR “early stage dementia”) AND (“spatial memory” OR “visuospatial memory” OR “spatial working memory” OR “visuospatial working memory” OR “visuospatial sketchpad”) was entered to search for studies providing analytical cross-sectional data about comparisons between MCI patients and reference population/s (e.g., healthy controls, patients with dementia) on tasks assessing VSWM abilities; the string (“mild cognitive impairment” OR “mci” OR “mild dementia” OR “early stage dementia”) AND (“anosognosia” OR “awareness”) AND (“memory”) was entered to search for studies providing analytical cross-sectional data about comparisons between MCI patients and reference population/s on tasks assessing anosognosia for memory deficits, and the string (“mild cognitive impairment” OR “mci” OR “mild dementia” OR “early stage dementia”) AND (“motor” OR “visuomotor” OR “movement*” OR “coordination” OR “eye-hand*”) was entered to search for studies providing analytical cross-sectional data about comparisons between MCI patients and reference population/s on tasks assessing visuomotor abilities. To search for studies providing longitudinal data (baseline and follow-up) about the progression from MCI towards dementia, we added the search term “conversion” OR “progression” to the abovementioned strings individually entered. The search was last updated on May 10, 2021.

Eligible studies were peer-reviewed written-in-English articles published in academic journals. Conference proceedings, letters to the editor, theses, commentaries, studies on animals, single-case studies, reviews, and studies not reporting (or partially reporting) the data of our interest were excluded. When two or more papers provided data from the same study population/s, the first paper in order of publication was selected. Since PMC hypometabolism is largely considered an AD-like pattern regardless of the specific MCI clinical subtype [10, 11], no restrictions were made on the basis of the MCI diagnosis.

### Data extraction, results, and synthesis

The first author and a research assistant (C.S.) performed a first screening based on titles and abstracts. Next, the first author, T.I., and A.I. independently performed the full-text assessment and selected the article to be included in the review.

The systematic search yielded a total of 20,136 articles. More specifically, the search strings identified 4,383 eligible articles investigating MCI patients’ performance on VSWM tasks, 578 eligible articles investigating patients’ performance on tasks assessing anosognosia for memory deficits, and 11,488 eligible articles investigating patients’ performance on tasks assessing visuomotor abilities. As concerns the systematic search for longitudinal studies, the search strings identified 3,687 articles, i.e., 712, 120, and 2,855 papers potentially exploring the predictive value of VSWM, anosognosia for memory deficits and visuomotor control, respectively.

Out of a total of 20,136 studies, 20,088 were removed according to the exclusion criteria and, additionally, if they (a) assessed visuomotor abilities in patients with MCI in Parkinson’s Disease (MCI-PD) because of the major motor impairment or (b) provided information about upper extremity functioning by self-report measures. The final set, therefore, consisted of 48 studies assessing the following issues: 16 studies assessed VSWM abilities [17, 25–39], 12 studies assessed anosognosia for memory deficits [40–51], 14 studies assessed visuomotor abilities [52–65], 2 studies investigated the relationship between VSWM and conversion [37, 66], and 4 studies investigated the relationship between anosognosia for memory deficits and conversion [67–70]. Based on our systematic search, studies examining the relationship between visuomotor impairment and conversion to dementia are not available.

As for cross-sectional data, the following information from the included articles was extracted: first author’s last name, publication year, country, study population/s and sample size, main assessment method and outcome variable/s, and results of the between-group comparison (see Table 1). As for longitudinal data on disease progression, we extracted data including first author’s last name, publication year, country, study population/s and sample size, main assessment method, main outcome variable/s measured at baseline, mean follow-up (in months), proportion of converted vs non-converted patients, results of between-group comparison conducted at baseline on the variable of interest, number of MCI patients who converted and were compromised at baseline, and conversion outcome (i.e., type of dementia; see Table 2). We summarized our findings narratively.

**Table 1:** Summary of included articles providing cross-sectional data

Authors	Country	Samples characteristics	Main assessment	Main outcome variable/s	Results
*Visuospatial* *Working* *Memory* (*n* = 16)					
Alichniewicz et al. (2012) [25]	Regensburg (Germany)	39 aMCI, 24 HCs	N-Back Task	Judgment accuracy	HCs > MCI
				Reaction time	HCs = MCI
Blatt et al. (2014) [26]	Magdeburg (Germany)	18 aMCI, 25 HCs	Task description: subjects had to remember the location of vertical bars and ignore horizontal bars	Storage ability	HCs = MCI
				Filtering ability	HCs > MCI
Caffò et al. (2012) [27]	Bari (Italy)	51 aMCI, 53 HCs	Reorientation paradigm	Number of attempts	HCs > MCI
de Rover et al. (2011) [28]	Cambridge (UK)	15 aMCI, 16 HCs	CANTAB-PAL Test	Recall accuracy	HCs > MCI
Elosúa et al. (2017) [29]	Madrid (Spain)	30 AD, 30 MCI [NOS], 30 HCs	Forward CBT (with inhibition)	Recall accuracy	HCs > MCI, HCs > AD
Kessels et al. (2010) [30]	Nijmegen (Netherlands)	15 aMCI, 25 HCs	Box task	Within-search errors	HCs = MCI
				Between-search errors	HCs > MCI
Kessels et al. (2015) [31]	Nijmegen (Netherlands)	14 AD, 11 MCI [NOS], 25 HCs	Backward CBT	Recall accuracy	HCs > MCI = AD
Kochan et al. (2010) [17]	Sydney (Australia)	35 MCI [NOS], 22 HCs	DMS task	Recall accuracy	HCs = MCI
				Reaction time	HCs = MCI
Lou et al. (2015) [32]	Hong Kong (China)	17 aMCI, 19 HCs	DMS task	Recall accuracy	HCs > MCI
				Reaction time	HCs = MCI
Maki et al. (2010) [33]	Maebashi (Japan)	27 PwD (18 AD, 6 LBD, 3 NOS), 10 MCI, 29 HCs	Numerical memory span task	Recall accuracy	HCs > MCI > PwD
Mitolo et al. (2013) [34]	Padua (Italy)	20 MCI [NOS], 14 HCs	OLRT	Accuracy in object recall and positioning	HCs > MCI
			MLT	Difference between observed and correct responses	HCs > MCI
Moodley et al. (2015) [35]	Haywards Heath (UK)	11 AD, 21 MCI [NOS], 20 HCs	4MT	Allocentric spatial memory	HCs > MCI > AD
Moodley et al. (2015) [35]	Milan (Italy)	9 AD, 14 MCI [NOS], 10 HCs	4MT	Allocentric spatial memory	HCs > MCI, HCs > AD, MCI = AD
Ruggiero et al. (2018) [36]	Naples (Italy)	8 AD, 11 aMCI, 19 HCs	EAT	Egocentric memory	HCs > AD, HCs = MCI, MCI = AD
				Allocentric memory	HCs > MCI, HCs > AD
				Egocentric-allocentric switching	HCs > AD, HCs = MCI, MCI = AD
				Allocentric-egocentric switching	HCs > MCI, HCs > AD
Ruggiero et al. (2020) [37]	Naples (Italy)	8 AD, 10 aMCI, 20 HCs	EAT	Egocentric-categorical memory	HCs = MCI, HCs > AD, MCI = AD
				Egocentric-coordinate memory	HCs = MCI > AD
				Allocentric-categorical memory	HCs = MCI > AD
				Allocentric-coordinate memory	HCs > MCI = AD
Ung et al. (2020) [38]	Perak (Malaysia)	18 AD, 12 MCI [NOS], 31 HCs	VWMT	Recall accuracy	HCs = MCI > AD
Wiechmann et al. (2011) [39]	Fort Worth (USA)	261 AD, 107 VaD, 55 aMCI, 71 naMCI, 44 HCs	Backward CBT	Recall accuracy	HCs > VaD, HCs > AD, HCs = aMCI = naMCI, aMCI > AD, naMCI > AD, naMCI > VaD
*Awareness* *of* *Memory* *Deficits* (*n* = 12)					
Chudoba & Schmitter-Edgecombe (2020) [40]	Washington (USA)	26 aMCI, 26 HCs	Study and recall of words from a 12-word list	OJD	HCs = MCI
Clare et al. (2013) [41]	Bangor (UK)	99 PwD (AD, VaD, AD + VaD), 30 MCI (aMCI and aMCI-md)	MARS-MFS	SRD	MCI = PwD
			MARS-MPS/RBMT	SRD + OJD	MCI > PwD
Coutinho et al. (2016) [42]	Rio de Janeiro (Brazil)	22 MCI (aMCI and aMCI-md), 25 HCs	RAVLT (5^th^ trial) vs MAC-Q	Discrepancy between objective and subjective memory measures	HCs = MCI
Fragkiadaki et al. (2016) [43]	Athens (Greece)	35 aMCI, 35 HCs	HVLT-Delayed Recall	OJD	HCs > MCI
			BVMT-Delayed Recall	OJD	HCs > MCI
Galeone et al. (2011) [44]	Naples (Italy)	15 AD, 25 aMCI, 21 HCs	AMIS	SRD	HCs > MCI = AD
			Study and recall of words from three 10-word lists	OJD	HCs > MCI = AD
Lehrner et al. (2015) [45]	Vienna (Austria)	43 AD, 137 aMCI, 181 naMCI, 211 HCs	FAI vs VRST-delayed recall	Discrepancy between objective and subjective memory measures	HCs > naMCI > aMCI > AD
Oba et al. (2018) [46]	Kyoto (Japan)	118 AD, 47 aMCI, 17 HCs	Questionnaire on anosognosia for memory impairment	SRD	HCs > MCI > AD
Ryu et al. (2020) [47]	Seoul (Republic of Korea)	49 AD, 51 MCI [NOS]	SMCQ	SRD	MCI = AD
Seelye et al. (2010) [48]	Pullman (USA)	27 aMCI, 14 naMCI, 41 HCs	RAVLT-delayed recall	OJD	HCs = aMCI = naMCI
Tondelli et al. (2018) [49]	Modena (Italy)	12 AD, 15 aMCI	RAVLT-Delayed Recall	OJD	MCI > AD
			ROCF-Delayed Recall	OJD	MCI > AD
Vannini et al. (2017) [50]	Boston (USA)	31 MCI (aMCI and aMCI-md), 251 HCs	Logical Memory-delayed recall (WMS-R) vs MFQ-general frequency of forgetting subscale	Discrepancy between objective and subjective memory measures	HCs > MCI
Vogel et al. (2004) [51]	Copenhagen (Denmark)	36 AD, 30 aMCI, 33 HCs	ARS	CR	MCI = AD
			SRSMF	SRD	HCs > MCI = AD
*Visuomotor* *Control* (*n* = 14)					
Aggarwal et al. (2006) [52]	Chicago (USA)	60 AD, 198 MCI [NOS], 558 HCs	Pegboard test	Manual dexterity	HCs > MCI > AD
Camarda et al. (2007) [53]	Palermo (Italy)	11 AD, 11 aMCI, 11 HCs	Reaching task	Hand movement velocity	HCs = MCI > AD
Colella et al. (2021) [54]	Rome (Italy)	14 aMCI, 16 HCs	Finger-tapping task	Movement rhythm	HCs > MCI
				Movement amplitude	HCs = MCI
				Movement velocity	HCs = MCI
De Paula et al. (2016) [55]	Belo Horizonte (Brazil)	38 AD, 34 aMCI, 32 aMCI-md, 20 HCs	Nine-hole peg test	Manual dexterity	HCs = aMCI, HCs > aMCI-md, HCs > AD; aMCI = aMCI-md, aMCI > AD, aMCI-md = AD
Franssen et al. (1999) [56]	New York (USA)	101 AD, 69 MCI [NOS], 195 HCs	Sequential finger to thumb tapping task	Manual coordination	HCs > MCI > AD
Huang et al. (2019) [57]	Taipei (Taiwan)	36 AD, 43 MCI [NOS], 41 HCs	Spiral examination	Hand movement velocity	HCs = MCI = AD
			Yong examination	Hand movement trajectory	HCs > MCI > AD
				Hand movement velocity	HCs > MCI > AD
Kluger et al. (1997) [58]	New York (USA)	25 AD, 25 MCI [NOS], 41 HCs	Finger-tapping task	Gross motor skills	HCs = MCI > AD
			Purdue pegboard	Fine motor skills	HCs > MCI = AD
			Grooved pegboard	Fine motor skills	HCs > MCI = AD
			Purdue pegboard assembly test	Complex motor skills	HCs > MCI > AD
Roalf et al. (2018) [59]	Pennsylvania (USA)	131 AD, 46 MCI [NOS], 62 HCs	Finger-tapping task	Fine motor skills	HCs > MCI = AD
Robens et al. (2019) [60]	Tübingen (Germany)	56 AD, 64 MCI (aMCI and aMCI-md), 67 HCs	Digital tree drawing test	Hand movement velocity	HCs > MCI = AD
Salek et al. (2011) [61]	Toronto (Canada)	10 MCI (aMCI, aMCI-md, and naMCI-sd), 10 HCs	Visuomotor integration task	Hand movement velocity	HCs > MCI
Schröter et al. (2003) [62]	Munich (Germany)	35 AD, 39 MCI [NOS], 40 HCs	Handwriting task	Manual coordination	HCs > MCI > AD
Suzumura et al. (2018) [63]	Obu (Japan)	31 AD, 15 MCI [NOS], 48 HCs	Finger-tapping task	Finger Dexterity	HCs > MCI > AD
Yan et al. (2008) [64]	Hayward (USA)	9 AD, 9 aMCI, 10 HCs	Handwriting task	Hand movement velocity	HCs > MCI > AD
				Hand movement smoothness	HCs > MCI > AD
Yu et al. (2019) [65]	Kaohsiung (Taiwan)	22 AD, 14 aMCI, 18 HCs	Graphomotor Task	Hand movement accuracy	HCs > MCI = AD

>  Better Performance,  =  Similar Performance, *4MT* Four Mountains Test, *AD* Alzheimer’s Disease, *aMCI* Amnestic Mild Cognitive Impairment-Single Domain, *aMCI-md* Amnestic Mild Cognitive Impairment-Multiple Domain, *AMIS* Awareness of Memory Impairment Scale, *ARS* Anosognosia Rating Scale, *BVMT* Brief Visuospatial Memory Test, CANTAB-PAL Test, Cambridge Neuropsychological Test Automated Battery-Paired Associates Learning Test, *CBT* Corsi’s Block-Tapping Test, *CR* Clinician Rating, DMS Task, Delayed Match-to-Sample Task, *EAT* Ego-Allo Task, *FAI* Forgetfulness Assessment Inventory, *HCs* Healthy Controls, *HVLT* Hopkins Verbal Learning Test, *LBD* Lewy Body Dementia, *MAC-Q* Memory Complaint Questionnaire, *MARS-MFS* Memory Awareness Rating Scale-Memory Functioning Scale, *MARS-MPS* Memory Awareness Rating Scale-Memory Performance Scale, *MCI* Mild Cognitive Impairment, *MFQ* Memory Functioning Questionnaire, *MLT* Map Learning Test, *naMCI* Non-Amnestic Mild Cognitive Impairment-Single Domain, *naMCI-md* Non-Amnestic Mild Cognitive Impairment-Multiple Domain, *NOS* Not Otherwise Specified, *OJD* Objective Judgement Discrepancy, *OLRT* Objects and Location Recognition Test, *PwD* Patients with Dementia, *RAVLT* Rey Auditory Verbal Learning Test, *RBMT* Rivermead Behavioral Memory Test, *ROCF* Rey-Osterrieth Complex Figure, *SMCQ* Subjective Memory Complaint Questionnaire, *SRD* Subjective Rating Discrepancy, *SRSMF* Self-Rating Scale of Memory Functions, *VaD* Vascular Dementia, *VRST* Verbal Selective Reminding Test, *VWMT* Visuospatial Working Memory Task, *WMS-R* Wechsler Memory Scale-Revised

**Table 2:** Summary of included articles providing longitudinal data on conversion from MCI to dementia

Authors	Country	Sample	Main assessment at baseline	Main outcome variable/s at baseline	Follow-up in months, mean (SD)	Non-converted/converted	Non-converted vs converted on the baseline assessment	MCI-converted (unimpaired/impaired at baseline)	Type of dementia
*Visuospatial Working Memory (n=2)*									
Ruggiero et al. (2020) [37]	Naples (Italy)	10 aMCI	EAT	Allocentric-Coordinate Spatial Memory	NR	2/8	NR	1/7	AD
Wood et al. (2016) [66]	Falmer (UK)	15 MCI [NOS]	4MT	Allocentric Spatial Memory	24	6/9	Non-Converted > Converted	NR	AD
*Awareness of Memory Deficits (n= 4)*									
Bastin et al. (2021) [67]	Liège (Belgium)	44 MCI (39 aMCI, 5 aMCI-md)	MARS-MFS	SRD	21.40 (11.80)	21/23	Non-Converted > Converted	NR	AD
Munro et al. (2018) [68]	Boston (USA)	33 aMCI	Memory composite score vs MFQ-general frequency of forgetting subscale	Deviation of the objective from the subjective memory measure	19.10 (8.40)	23/10	Non-Converted > Converted	NR	AD
Nobili et al. (2010) [69]	Genoa (Italy)	42 aMCI	MAC-Q	Self-evaluation of memory decline	14.80 (6.40)	24/16 (2 drop-outs)	NR	9/7	14 AD, 1 FTD, 1 VaD
Spalletta et al. (2014) [70]	Rome (Italy)	36 aMCI	MIQ-memory subscale	SRD	60	26/10	Non-Converted > Converted	NR	AD

> , Better Performance, *4MT* Four Mountains Test, *AD* Alzheimer’s Disease, *aMCI* Amnestic Mild Cognitive Impairment-Single Domain, *aMCI-md* Amnestic Mild Cognitive Impairment-Multiple Domain, *EAT* Ego-Allo Task, *FTD* Frontotemporal Dementia, *MAC-Q* Memory Complaint Questionnaire, *MARS-MFS* Memory Awareness Rating Scale-Memory Functioning Scale, *MCI* Mild Cognitive Impairment, *MFQ* Memory Functioning Questionnaire, *MIQ* Memory Insight Questionnaire, *NOS* Not Otherwise Specified, *SRD* Subjective Rating Discrepancy, *VaD* Vascular Dementia

## MCI and visuospatial working memory

By definition, the VSWM is responsible for the brief-term retention and “online” manipulation of spatial items without any extrinsic facilitation [71]. From a neurofunctional standpoint, it has been suggested that visuospatial material is initially stored in the posterior parietal cortex (PPC) and then it is projected into frontal areas where it is manipulated in line with the task demands [72]. There is experimental evidence supporting this hypothesis [18, 25, 28]. For instance, Luber et al. [18] showed that the application of a repetitive transcranial magnetic stimulation (rTMS) at 5 Hz to the parietal site, centered on the precuneus, during the performance of a VSWM task, significantly reduced reaction times, net of accuracy. This finding suggests a clear involvement of posteromedial parietal regions in the short-term maintenance and manipulation of spatial information.

VSWM abilities have been assessed in patients with MCI [17, 25–39] (Table 1). In the experiment by Alichniewicz et al. [25], the authors employed a paradigm based on the n-back principles to test VSWM in patients with aMCI and healthy participants. The n-back task, originally designed to test WM on verbal material, is one of the most powerful and reliable experimental paradigms used to properly assess WM functioning [73]. Typically, it requires the participant to respond when the currently presented item, within a stream of stimuli, is the same item presented “n” trials earlier, where “n” denotes the number of trials. The latter varies to increase the WM load, ranging from immediate repetition (0-back trials, control condition) to intervals of two (i.e., 2-back) or more items [74–76]. Alichniewicz et al.’s experiment included 2-back and 0-back trials. In the 2-back trials (i.e., the WM condition), participants were asked to monitor the color (red, green, yellow, blue) and location of series of dots and to indicate whether the currently presented stimulus was identical (same color and position) to the one presented two trials before. In the 0-back trials (i.e., the control condition), participants had to respond whenever a prespecified stimulus was presented (e.g., a red dot at a particular location). Although reaction times did not differ between the two groups, patients with aMCI performed significantly worse than controls in terms of accuracy. Such poor accuracy was associated with reduced activation in the middle frontal gyrus, superior parietal, and inferior parietal lobules [25]. In a subsequent study, where a hybrid n-back task was used, aMCI patients’ performance deteriorated with increasing memory load, i.e., passing by 1-back to 3-back condition [77]. This performance deterioration might be due to a PMC hypoactivation as the WM load increased, as showed by Kochan et al. [17]. In this functional magnetic resonance imaging (fMRI) study, patients with MCI and control subjects performed a visuospatial delayed match-to-sample task of increasing complexity that required providing “old-new” judgments in response to spatial configurations previously explored. More precisely, participants were asked to memorize a spatial configuration (i.e., in a 5 × 5 matrix) in which some quadrants were filled with abstract polychrome drawings (targets); the remaining quadrants were empty. The encoding phase lasted 6 s. When the learning time elapsed, a fixation mark appeared on the screen for 8 s. Thus, a new spatial configuration was displayed, and participants provided their judgment by pressing a button depending on whether at least one target was presented in the same position as the previous configuration. The authors found that activation in the posterior cingulate cortex and precuneus was inversely proportional to the cognitive/memory load in MCI patients compared with controls [17]. Interestingly, in a subsequent longitudinal study, the same research group showed that PMC deactivation, in patients with MCI, was a risk factor associated with worsening in functional autonomies, and therefore the quality of life, over 2 years [78].

Apart from the n-back and match-to-sample tasks, the most varied assessment procedures were used to test VSWM abilities in MCI, e.g., the classical Corsi’s block-tapping test [31, 39] and its variants [29], spatial search paradigms [30], tasks assessing the ability to recall the position of objects in space [28, 34, 38], to learn maps [34], or to formulate delayed spatial judgments according to specific frames of reference [35–37]. Overall, except for a few studies [17, 26, 38, 39], most of the available experimental evidence suggested that patients with MCI showed, on some level, a certain degree of VSWM deterioration, as indicated by the poorer performance than healthy controls [27–30, 32–35], which was often comparable to that of patients with AD [31, 36, 37] (Table 1).

Only two studies [37, 66] explored the possible relationship between VSWM and conversion from MCI to dementia. These will be discussed later in relation to the domain of spatial cognition.

## MCI and anosognosia for memory deficits

As stated by Andreasen et al. [16], the “resting brain” is “both active and interesting”. Accordingly, the authors measured cerebral perfusion of healthy subjects during the resting state by using positron emission tomography (PET). Participants were asked to remain silent and with their eyes closed, without providing any additional prompt. Results showed intense activation in a large corticocortical network including, more on the right side, frontal and parietal regions, precuneal and retrosplenial cortices, posterior cingulate cortex, and supramarginal and angular gyri [16]. Subsequent studies by Raichle et al. [21, 79, 80] provided converging evidence supporting the view that the brain works also, perhaps above all, in “background”, i.e., without supporting any specific task. In one of their experiment [21], PET was used to quantify the regional changes in the oxygen extraction fraction (OEF), i.e., the ratio between the brain’s oxygen consumption compared to the global oxygen delivery, during a baseline/resting state condition. In line with data provided by Andreasen et al., the authors circumscribed a neural network including the prefrontal cortex, precuneus, and the adjacent posteromedial parietal regions. These cortical territories, also known as “hot spots”, or “default mode network” (DMN) as a whole [15, 20], exhibited a significantly high metabolic activity in the resting state [21]. Gusnard and Raichle [79] suggested that the remarkable energy expenditure related to the high metabolic activity of DMN allows preserving the synaptic transmission functionality and promoting constant information processing. Intriguingly, the tonic activity of DMN attenuated when subjects performed perceptual or goal-directed non-self-referential cognitive tasks, with particular regard to precuneus, posterior cingulate cortex, and retrosplenial cortex [15, 20, 21]. These types of tasks elicited the so-called “task-induced deactivation” effect, which involved, in a specular way, the same areas showing increased activation at baseline. This functional dissociation is ascribable to the nature of information processed in the background. Accordingly, DMN is selectively recruited for assembling and updating information concerning the self and the surrounding world [15, 20]. Furthermore, DMN would appear to mediate several aspects of introspective cognition including mind-wandering, remembering the past, or anticipating the future [81]. A few years earlier, Andreasen et al. [16] already hypothesized that the cortical network they discovered, basically comparable to the DMN, could be involved in the interconnection between personal identity and autobiographical memory, thus constituting the possible neural substrate of self-awareness. In support of the existence of a strong relationship between DMN and self-awareness, aberrant activity in DMN has been often recorded in altered conscious states (e.g., slow-wave sleep, rapid eye movement sleep, general anesthesia, drug-induced coma, hypnosis) or in some neuropsychiatric conditions characterized by impaired awareness such as AD, autism, schizophrenia, and epilepsy [19, 20, 82].

Abnormal DMN activity has also been observed in patients with aMCI [81, 83–89]. Some studies reported a decreased activation of the posterior cingulate cortex and precuneus in aMCI patients undergoing resting-state fMRI compared with healthy subjects [81, 83, 86, 89]. Furthermore, MCI patients showed decreased functional connectivity in the posterior cingulate cortex and precuneus [90–92] (see also Eyler et al. [93] for a review). Finally, a reduced PMC deactivation was recorded in aMCI patients engaged in cognitive tasks. Loss of PMC deactivation appears to have a strong prognostic significance since MCI patients showing lower PMC deactivation are more likely to convert to dementia over about 3 years [85]. Although an awareness alteration is not traditionally considered a pathognomonic symptom of MCI, these results tend to suggest that a deficit of awareness might be of significant interest for MCI nosographic classification.

From a neuropsychological standpoint, the notion of “lack of awareness” is closely linked to the phenomenon of anosognosia referring to the inability of patients to recognize their own deficits. This phenomenon has important clinical implications, since the patient’s (or relatives’) tendency to underestimate symptoms or to ascribe them to the mere aging may delay the request for a well-timed clinical consultation or affect therapy adherence [94], besides being associated with higher caregiver burden [95].

There are different methods for measuring anosognosia. The Subjective Rating Discrepancy (SRD), also known as “patient-informant discrepancy”, requires both patient and her/his caregiver to rate patient’s symptoms/abilities by filling in two parallel versions of the same questionnaire. The discrepancy score is usually obtained by subtracting the informant’s rating from the patient’s rating. The Objective Judgement Discrepancy (OJD) requires the patient to provide a judgment concerning her/his performance immediately prior to (prediction) or following the (estimation) execution of a certain task. This estimation is compared with the actual performance. The Clinician Rating (CR) is based on the judgment of the clinician exploring the patient’s level of anosognosia through both clinical interviews and standardized assessment tools [49, 96].

These methods have some limits. For instance, the validity of SRD relies on the assumption that caregivers are reliable informants, and this can be questioned; indeed, their ratings might be influenced by a number of factors such as caregiver burden and frequency of time spent with the patient. Still, although the CR is the most widely used assessment method in the outpatient clinical practice, it might be too unspecific, and it may therefore fail to accurately describe the extent of the alleged metamemory deficits [51]. In the face of these limitations and clinically speaking, the assessment methods based on SRD and OJD are the most interesting since they appear to tap different dimensions of anosognosia. The SRD tests the integrity of an “offline” awareness architecture fed by the accumulated experience-based knowledge concerning cognitive successes or failures. Conversely, the OJD tests the integrity of an “online” awareness flow allowing to detect contingent cognitive successes or failures by monitoring the ongoing performance. As some studies reported, SRD scores showed no correlation with OJD scores [44, 96]. These results are consistent the Cognitive Awareness Model (CAM), one of the best-known theoretical-interpretative models of anosognosia, which ascribes anosognosia to a multidimensional construct where selective impairments might cause different types of awareness deficits [96].

According to the Petersen’s criteria [1–4], the diagnosis of MCI should be carried out in the presence of subjective cognitive complaints reported by the patient and preferably corroborated by a caregiver, and successively confirmed by neuropsychological assessment, in the absence of gross cognitive and functional decline such as to delineate a clinical picture of dementia. However, some studies showed that aMCI patients demonstrated poor awareness of their memory deficits [41, 43–47, 50, 51] (Table 1). Therefore, the presence of subjective complaints might not be a mandatory prerequisite for the clinical diagnosis of MCI [43, 51].

Vogel et al. [51] employed both the CR and the SRD for comparing the level of awareness of memory deficits of aMCI patients, mild AD patients, and healthy subjects. Anosognosia for memory deficits was detected in both aMCI and AD patients, regardless of the assessment method used. Moreover, the authors found no statistically significant difference between aMCI and AD in terms of awareness. As a consequence, they suggested that anosognosia for memory deficits could be equally frequent in aMCI and overt AD [51]. Later studies using similar assessment methods reached the same conclusions [41, 44, 47]. In particular, in the experiment by Galeone et al. [44], aMCI, AD patients, and control healthy subjects were tested by using both SRD- and OJD-based methods. The SRD score was obtained through the administration of a brief questionnaire, derived from Ansell and Bucks [97], exploring the presence of daily life memory failures. Both participants and their caregivers completed the questionnaire to obtain the discrepancy score. To calculate the OJD score, participants were instead asked to make predictions on their memory performance, namely, recalling items from three 10-word lists. The experimental procedure was as follows. First, participants completed the questionnaire. Second, they were informed that a 10-word list would have been presented; then, participants were asked to predict how many words they would have been able to recall (pre-study prediction). Subsequently, they read each word aloud. After the encoding session, participants were required to provide a further prediction (post-study prediction) and then recalled the words of the list. This procedure was the same for each of the three lists. After the third list, participants completed the questionnaire again. Results showed that both MCI and AD patients demonstrated decreased awareness of memory deficits (SRD) compared with control participants and did not revise their rating following exposure to the memory task. Furthermore, MCI and AD patients consistently overestimated their performance (OJD) across the three lists, whereas controls progressively revised their prediction so that, at the third list, it was substantially optimal [44]. Interestingly, patients with aMCI appear to overestimate their memory performance even if tested on visuospatial material [43]. The observation of similar patterns of impaired awareness in MCI and patients with dementia (in particular with AD) supports the view of anosognosia as a continuum between the prodromal stage and overt dementia [44]. It is important to note that other studies using procedures based on the OJD observed no difference between patients with aMCI and healthy controls [40, 48]. Some methodological (e.g., the sample size and the neuropsychological tool used) and statistical issues may account for these conflicting results.

As concerns the neurofunctional correlates of anosognosia for memory deficits, it has been recently proposed that decreased connectivity in the fronto-temporo-parietal network, including the medial temporal lobe, would prevent updating of the state of memory functioning, which depends on the experience-based knowledge accumulated through the continued exposure to memory successes or failures. Conversely, abnormal activity in medial-frontal and -parietal regions would affect online monitoring and evaluation of cognitive performance [67]. Recent neuroimaging studies showed that greater anosognosia for memory deficits in amnestic MCI was associated with (1) hypometabolism in posterior and middle cingulate cortices, inferior parietal lobule, precuneus, angular gyrus [50, 69], hippocampus [49, 50], and (2) reduced functional connectivity between the posterior cingulate cortex, orbitofrontal cortex and inferior parietal lobe [50]. In line with these observations, it has been observed that patients with aMCI and anosognosia for memory deficits exhibited hypometabolism in the precuneus, inferior parietal lobule, superior occipital, angular, and middle temporal gyri compared with healthy controls [69].

Four studies [67–70] tested the hypothesis that anosognosia for memory deficits represented a risk factor for conversion to dementia (Table 2). The results of three out of the four studies [67, 68, 70] clearly supported this hypothesis. By bringing together the available data collected on a population of 153 patients with MCI (148 aMCI and 5 aMCI-md), 59 patients (38.56%) converted to dementia (57 AD, 1 FTD, 1 VaD) within about 2 years. On average, MCI patients who converted demonstrated poorer awareness of their memory deficits at baseline compared with non-converters.

## MCI and visuomotor control

The PPC occupies an anatomically strategic position allowing it to integrate and process multisensory afferent signals from anterior-parietal (e.g., primary somatosensory cortex) and cortico-subcortical motor areas. It has anatomo-functional relationships with basal ganglia and cerebellum, which are responsible for the regulation of sequential movements’ amplitude and velocity, and for planning and execution of fine and coordinated movements, respectively. PPC is strictly involved in eye-hand coordination, e.g., to reaching an object under visual guidance, in the real-time correction of goal-directed reaching movements, and unimanual and bimanual coordination of reach-to-grasp movements [15, 74, 98, 99]. Hence, it plays a pivotal role in planning, initiating, and executing hand movements. The reaching efficiency depends on (1) the integrity of both feedforward and feedback-based mechanisms, which regulate planning and in-flight proprioception/sight-based corrections, respectively [100–102]; and (2) the bottom-up processing of spatial coordinates characterizing the metric relationships between agent and target object. The latter leads to the construction of spatial representations based on the egocentric frame of reference within an occipitoparietal-frontal network [103]. The egocentric frame of reference serves the body (or body parts) as an “anchor” for computing the position of an object in space. Its integrity is necessary for planning and controlling visually-guided movements in the reaching space. The allocentric frame of reference, instead, defines the position of an object in relation to other objects present in the environment, independently of the observer’s perspective. The use of allocentric coordinates is needed during the execution of memory-driven actions [104, 105]. Although allocentric spatial coding is related to activity in the parietal cortex, it requires an additional involvement of the occipito-temporal stream to work properly [103, 105].

Suggestive neuropsychological observations support the view that visually-guided hand movements are mainly “mounted” in the PMC [23]. It is well known that parietal lobe injuries may result in optic ataxia, namely, an impairment in performing hand movements to reach and grasp a visually-presented object [105]. A patient with unilateral parietal damage and optic ataxia generally shows marked impairment when required to reach an object presented in her/his contralesional/ataxic visual field (“field effect”) and also when required to use her/his contralesional/ataxic hand (“hand effect”). However, patients with optic ataxia may not show any reaching difficulties when the target object is centrally presented and correctly foveated [106]. Karnath and Perenin [24] analyzed the lesion pattern of 52 unilateral stroke patients, 16 of whom suffered from optic ataxia. In ataxic patients, they found a lesion overlap circumscribed to the PMC and centered on the human homologous of the “monkey’s parietal reach region”, i.e., the precuneus, close to the occipito-parietal junction [24]. Converging evidence comes from other neuroimaging studies suggesting a clear involvement of the precuneus in planning and executing reaching movements [22, 23]. Interestingly, as recently speculated [107], the anatomofunctional changes observed in the precuneus of MCI patients, and the link between the precuneus and optic ataxia, suggest that optic ataxic misreaching might be detectable in patients with MCI, particularly for objects in peripheral vision.

So far, visuomotor abilities in MCI received little attention in terms of diagnostic/prognostic significance, probably since apraxic/ataxic phenomena and gross movement impairments are traditionally classified as clinical signs of an intermediate/advanced stage of a major neurocognitive disorder. In support of this claim, some studies found no difference in terms of hand movement kinematics (i.e., speed, dexterity, movement amplitude) between MCI patients and healthy controls [53–55, 57, 58]. However, the majority of data available in the literature suggests that visuomotor abilities are compromised in MCI [52, 54–65] (Table 1). Particularly, hand movements of MCI patients were found to be less accurate [57, 65], slower [57, 60, 61, 64], and less coordinated [52, 54–56, 58, 59, 62, 63] as compared with healthy controls. Some studies suggested, moreover, a similar degree of visuomotor deterioration in patients with MCI and AD [55, 58–60, 65].

Different types of tasks were used to test visuomotor abilities in MCI, e.g., the Pegboard test and its variants [52, 55, 58], the finger-tapping task [54, 56, 58, 59, 63], reaching tasks [53], visuomotor integration tasks [61], and handwriting/graphomotor tasks [57, 60, 62, 64, 65]. For instance, Kluger et al. [58] administered to patients with AD, MCI, and to healthy controls an extensive battery for the assessment of psychomotor functions. The battery included tests of complex motor (e.g., Purdue pegboard assembly test), gross motor (e.g., finger-tapping speed, steadiness, strength), and fine motor control (i.e., Purdue pegboard and Grooved pegboard tests). The latter required unimanual or bimanual placement of pegs into precise locations on slotted boards. Intact eye-hand coordination is necessary to accurately perform these tasks. AD patients did significantly worse than controls in complex, gross, and fine motor tasks, whereas patients with MCI showed a simple dissociation, with spared gross motor and impaired complex and fine motor skills. Authors hypothesized that deficits in controlling visually-driven fine movements may occur well before gross motor impairment reaches the clinical observation [58]. In a later experiment, Yan et al. [64] tested fine visuomotor control skills in AD, aMCI, and healthy subjects by using a Fitts-like paradigm. Participants were instructed to hold a stylus with the dominant hand and to quickly move the stylus between two dots on a digitizer. Both aMCI and AD patients were slower, and less coordinated, compared with controls, and aMCI patients were faster, and more coordinated, compared with AD patients. In a recent study by Yu et al. [65], patients with AD, aMCI, and healthy participants were asked to copy some models (i.e., a square, a cross, the Chinese character “井”, and the Chinese character “正”) as accurately as possible. The results showed that accuracy in performing handwriting movements was significantly lower in the AD and aMCI groups than in the control group. Furthermore, no difference was found between patients with aMCI and AD.

Based on our systematic search, no study explored the relationship between visuomotor control and conversion from MCI to dementia. However, it has been speculated that the assessment of visuomotor abilities in individuals at high AD risk may be useful to discriminate patients who convert from those who do not convert to dementia [61, 64].

## Relationships with spatial cognition

PMC is also involved in spatial cognition, which is a multi-component system embracing several cooperating abilities needed for everyday activities, e.g., route finding, or locating a target position in space. Signs of topographical disorientation, i.e., the inability to find the way within large-scale environments, often follow PMC damage [108]. PMC acts in concert with temporal and frontal areas for processing egocentric and allocentric spatial information [15, 103–105], and it is widely acknowledged that the integrity of egocentric and allocentric representations is needed to spatially navigate in, and to act on, complex environments. Interestingly, spatial navigation disorders have been observed in patients with MCI, thus suggesting a possible impairment of spatial cognition in the prodromal stage of dementia (see Iachini et al. [109] for a review). It has been reported that impairment of allocentric spatial memory [37, 66] might represent an early predictor of conversion from MCI towards AD (Table 2).

In the study by Ruggiero et al. [37], the Ego-Allo Task (EAT) [110] was used. Eight patients with AD, 10 with aMCI, and 20 healthy controls were asked to memorize (6 s) the spatial position of three geometrical 3D objects placed in the peripersonal space. After a short delay (5 s), participants verbally provided one of four types of spatial judgments: egocentric-coordinate (e.g., “which object was closest/farthest to you?”), egocentric-categorical (e.g., “which object was on your left\right?”), allocentric-coordinate (e.g., “which object was closest/farthest to the cylinder?”), and allocentric categorical (“which object was on the left\right of the cylinder?”). Patients with aMCI demonstrated, at baseline, a selective decline in processing allocentric-coordinate information, arising from a more fine-grained analysis of between-object metric relationships. Follow-up data showed that 8/10 aMCI patients converted into AD.

In the longitudinal study by Wood et al. [66], the authors tested the hypothesis that a deficit in allocentric spatial memory was predictive of conversion from MCI to dementia. Fifteen patients with MCI underwent baseline testing on the Four Mountains Test (4MT), namely, a delayed match-to-sample-like task requiring participants to recall the spatial configuration of a series of computer-generated landscapes from a shifted viewpoint. More specifically, participants were presented with a sample image for 8 s. After a 2 s delay, the sample image was re-presented from a different viewpoint together with three distractors depicting similar landscapes. Participants were asked to indicate which of the four alternatives corresponded to a shifted view of the sample image. At 24 months, 9 out of the 15 patients converted to AD. The 4MT predicted conversion (93% accuracy), with MCI patients who converted obtaining lower performance at baseline compared with non-converters.

## Neuropsychological assessment of PMC

**Fig. 2:**
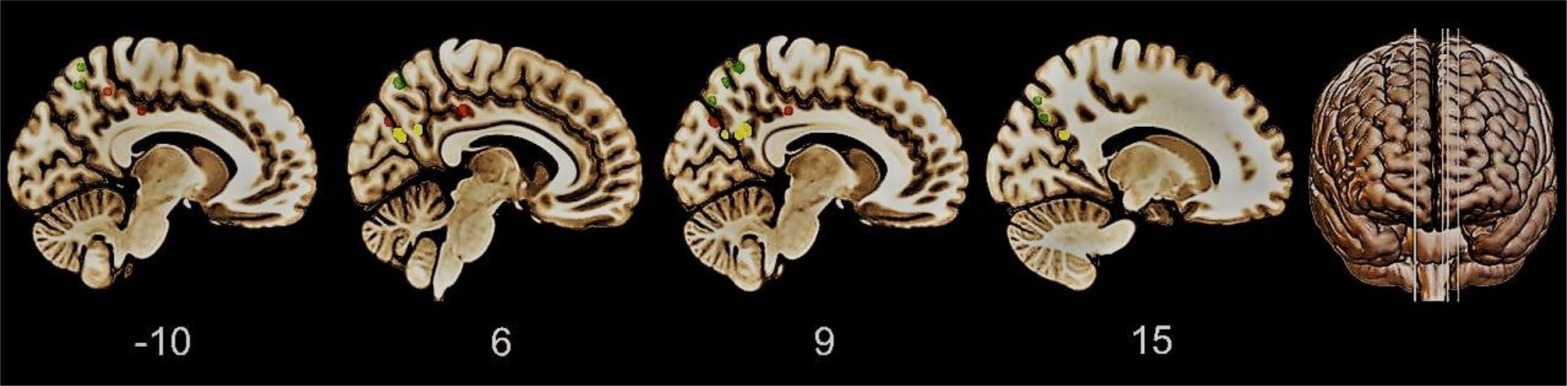
Graphical representation of the main PMC regions involved in visuospatial WM (yellow), awareness of memory deficits (red), and visuomotor control (green), i.e., posterior cingulate cortex (PCC) and precuneus (Pc). MRIcroGL software was used to circumscribe the ROIs on sagittal slices of T1-standard-template MRI. ROIs were extracted based on data from: Kochan et al. [17], Nobili et al. [69], Vannini et al. [50], and Karnath & Perenin [24]. Talairach coordinates were converted into MNI when necessary. MNI coordinates for visuospatial WM, PCC: *x* = 9, *y* = –57, *z* = 27; Pc: *x* = 6, *y* = –69 *z* = 24; *x* = 12, *y* = –60, *z* = 24. MNI coordinates for anosognosia for memory deficits, PCC: *x* = 6, *y* = –31, *z* = 38; *x* = –7, *y* = –29, *z* = 38; Pc: *x* = –7, *y* = –49, *z* = 49; *x* = 8, *y* = –74, *z* = 32. MNI coordinates for visuomotor control, Pc: *x* = –8, *y* = –64, *z* = 63; *x* = –8, *y* = –68, *z* = 53; *x* = 6, *y* = –70, *z* = 54; *x* = 7, *y* = –68, *z* = 54; *x* = 10, *y* = –60, *z* = 63; *x* = 13, *y* = –73, *z* = 44; *x* = 18, *y* = –70, *z* = 34

There is evidence of abnormal PMC activity underpinning deficits in VSWM [17] and awareness of memory abilities [50, 69] in MCI. Visuomotor skills appear also compromised in patients with MCI [52, 56–65], although neuroimaging evidence binding visuomotor deficits to PMC dysfunction are not currently available for this cohort of patients. Nevertheless, as the visuomotor domain partially shares the same neural correlates of VSWM and awareness of memory deficits (Fig. 2), it is reasonable to hypothesize that dysfunction of PMC may also account for visuomotor deficits in patients with MCI [53, 61, 107]. The presence of deficits in some or all three domains may suggest an early PMC alteration which has been, in turn, associated with a higher risk of conversion from MCI to AD [9–11]. Furthermore, VSWM and metamemory deficits were found to be individually and significantly associated with a higher risk of conversion [37, 66–70]. Consequently, their clinical importance is not at all negligible.

To evaluate the integrity, or detect the impairment, of these neurocognitive domains, the use of easy and quick-to-administer tasks could constitute a precious help for clinicians. Here, we report some tests that we suggest being, at the same time, easy-to-use and sensitive enough to assess VSWM, awareness of memory deficits, and visuomotor abilities during outpatient clinical practice.

### Assessment of visuospatial working memory

*Mental Rotations Test:* [111] It demands to identify, within a set of pictorial stimuli, which stimulus corresponds to a rotated view of the criterion/target figure. Although the test is conventionally used to assess mental visualization abilities, it involves kinds of elaborations fitting closely with current conceptions of WM. Indeed, to mentally rotate an object, subjects generate and actively maintain the object’s mental representation and the interrelations of its component parts, and continuously update the representation while the object rotates. By interposing a short delay between the presentation of the target figure and the response set, mental rotation should further burden WM capacity.

*Backward Corsi’s Block-Tapping Test:* [112] The apparatus consists of nine irregularly arranged blocks. The examiner taps the blocks, one per second, based on given sequences increasing in length. The subject is required to repeat the tapping sequence in reverse order.

*Corsi’s Block-Tapping Test with inhibition:* [29] This task is similar to the standard forward Corsi’s test, except that participants must inhibit, and not repeat, the second block of each tapping sequence.

*Jigsaw-Puzzle Imagery Task* [113] The material comprises pictures depicting common objects. Each picture is fragmented into some numbered pieces, like a puzzle. Subject is asked to mentally rebuild the puzzle by writing the pieces’ numbers on a blank matrix in the correct spatial positions.

*Delayed-Response-Activity Test (Stanford–Binet Intelligence Scales–Fifth Edition, SB5)* [114] The task is quite similar to the so-called “three-card trick”. The examiner hides a little ball underneath one of the two (or three) cups in front of the subject. Subsequently, the examiner rearranges the cups always in plain view of the subject. She/he is asked to indicate the cup in which the little ball has been hidden.

*Pathway Span Task* [115] It demands to mentally visualize and maintain in memory the path followed by a little man moving on a blank matrix. The task complexity depends on the size of the matrix and the length of the path.

### Assessment of anosognosia for memory deficits

A comprehensive protocol to assess anosognosia for memory deficits should include both SRD- and OJD-based methods, combined with the CR, if necessary.

#### Subjective rating discrepancy

*Self-Rating Scale of Memory Functions (SRSMF)* [116] This is an 18-item self-report questionnaire originally constructed to detect subjective memory complaints in hospitalized psychiatric patients with depression. Patients were required to compare their present memory abilities to the same abilities before hospitalization.

*Memory Observation Questionnaire (MOQ)* [117] It is composed of 20 items exploring self-perception of the current memory functioning.

*Memory Complaint Questionnaire (MAC-Q)* [118] This brief 6-item questionnaire explores the self-perception of age-related memory decline. Subject is asked to rate her/his memory abilities as compared to when she/he was in high school or college.

*Metamemory Questionnaire–Ability Subscale (MMQ-A)* [119] It is a 20-item questionnaire assessing awareness of memory failures in everyday life. The patient is asked to indicate the frequency with which she/he made memory mistakes over the past two weeks.

*Subjective Memory Complaint Questionnaire (SMCQ)* [120] The questionnaire consists of 14 items exploring both global and daily memory functioning.

For each of the aforementioned tools, note that a parallel version of the questionnaire has to be completed by a caregiver to obtain the discrepancy score.

#### Objective judgement discrepancy

The experimental procedure drawn by Galeone et al. [44] appears straightforward and relatively fast. Consequently, it could be declined in the clinical practice as a valid OJD-based assessment method.

#### Clinician rating

*Anosognosia Rating Scale (ARS)* [121] It is a semi-quantitative clinical instrument to quickly assess the degree of awareness of memory deficits. The examiner interviews the subject who subsequently undergoes a neuropsychological examination. Then, the subject is rated on a 4-point scale: full awareness, shallow awareness, no awareness, or denies impairment.

*Clinical Insight Rating Scale (CIRS)* [122] Based on separate interviews with both patient and her/his caregiver, this scale explores four domains of the patient’s awareness, namely, the reason for the visit, cognitive deficits, functional deficits, and progression of deficits.

*Experimenter Rating Scale (ERS)* [96] This is a 4-point scale allowing the clinician to rate the degree of anosognosia for memory deficits according to the extent to which the subject reports memory difficulties.

### Assessment of visuomotor control

Note that we borrowed some tests from the literature on the optic ataxia and visuomotor integration deficits in developmental age. Validation and normative studies on older adults would guarantee higher reliability of the assessment procedure and results.

*Tapping and Dotting Subtests (MacQuarrie’s Test for Mechanical Ability, MTMA)* [123] These tasks measure speed and accuracy of eye-hand coordination. For both tasks, subject is provided with a test booklet and a pencil. The Tapping test requires subject to put three pencil dots in each printed circle as fast as possible within 30 s. The number of circles containing at least three dots is counted. The Dotting test requires, instead, to put only one pencil dot in each printed circle as fast as possible within 30 s.

*Purdue Pegboard Test* [124] It is a test of manual dexterity and coordination. The subject is required to put in the pegboard’s holes as many pins as possible within 30 s using the right or left hand, or both.

*Kas’ test* [125] This test was devised for studying eye-hand behavior in patients with optic ataxia. The subject sits in front of the examiner and is asked to fix the examiner’s nose (fixation point). The examiner moves a target object (e.g., a coin) through the four visual quadrants. The subject is therefore asked to touch the object without moving her/his gaze from the fixation point.

*Visual-Motor Speed and Precision Test (VMSPT)* [126] This is a practical, elegant, and very fast pencil-and-paper task designed to quantify the speed and precision of fine visuomotor coordination in children. The subject is presented with a sheet of white paper on which several little circles are printed. Proceeding from the top down, the circles decrease in size. The subject is instructed to make a cross completely inside each circle, one right after the other, as fast as possible, and without skipping any circle.

*Movement Assessment Battery for Children–Second Edition (MABC–2)* [127] This neuropsychological battery was traditionally intended to support the diagnosis of motor impairment in children. It consists of 24 motor tasks exploring manual dexterity, aiming/catching abilities, and balance.

*Eye-Hand Coordination Subtest (Developmental Test of Visual Perception–Third Edition, DTVP-3)* [128] It has been conceived for studying eye-hand coordination in children. The task demands to draw precise straight or curved lines as accurately as possible and staying within established visual boundaries.

## Conclusions and future directions

Clinical neuropsychology is traditionally considered a discipline using psychometric techniques to quantify, for diagnostic purposes, the extent of cognitive deficits following a neurological illness. However, searching for “neuropsychological indexes” sensitive enough to clinical progression to dementia could improve the prognostic accuracy and the quality of MCI patient management.

PMC hypoactivation appears to be significantly associated with an increased conversion rate from MCI to AD [9–11]. PMC is involved in VSWM [18], metamemory [50, 67, 69], and visuomotor control abilities [22–24]. Testing the integrity of this neuropsychological cluster could help clinicians to early discriminate MCI patients who are more likely to convert in probable AD from those who are not [12–14], thus orienting further investigations and/or preventive interventions.

To date, no intervention has proven effective in ultimately preventing conversion to dementia [129]. However, treatment with cholinesterase inhibitors seems to delay the conversion although not affecting the natural history of disease; lifestyle modifications including diet, aerobic exercise, and cognitive stimulation may decrease the risk of conversion [130]. Clinicians should consider anxiety [131, 132], apathy [133–135], diabetes [136], sleep [137, 138] and cardiovascular disorders [139] as other factors increasing the risk of conversion and negative outcomes.

Future investigations are needed to test the clinical value of the circumscribed neuropsychological assessment we proposed, and to establish whether the evaluation of some or all three domains, alone or in combination with instrumental investigations, actually increases the prognostic accuracy.
